# Supplementary material for: Effects of perinatal fluoxetine exposure on novelty-induced social and non-social investigation behaviors in a seminatural environment
Source: Psychopharmacology (Berl). 2021 Sep 23;238(12):3653–67. doi: 10.1007/s00213-021-05984-8 (PMC8629781; doi:10.1007/s00213-021-05984-8)
Supplement: Supplementary file 1 — (PDF 242 kb) [file 213_2021_5984_MOESM1_ESM.pdf]

Supplemental Materials

Effects of Perinatal Fluoxetine Exposure on Novelty-induced Social and Non-Social Investigation Behaviors in a Seminatural Environment

Ole Christian Sylte[1,*], Jesper Solheim Johansen[1,*], Indrek Heinla[1], Danielle J Houwing[1,2], Jocelien DA Olivier[2], Roy Heijkoop[1], and Eelke MS Snoeren[1,3]

[1] Department of Psychology, UiT the Arctic University of Norway, Norway

[2] Department of Neurobiology, Groningen Institute for Evolutionary Life Sciences, University of Groningen, the Netherlands

[3] Regional Health Authority of North Norway

[*] These authors contributed equally.

Corresponding concerning this article should addressed to Eelke Snoeren, Department of Psychology, UiT the Arctic University of Norway, 9037 Tromsø, Norway. E-mail address: eelke.snoeren@uit.no

Table S1

Treatment Dams and Cage Distribution Offspring

Dams	Treatment	Number offspring	Male-Cage 1	Male-Cage 2	Female-Cage 3	Female-Cage 4	Female-Cage 5	Female-Cage 6
F1	FLX	7	OM1<br>OM2<br>OM3		OF2<br>OF3	OF1<br>OF4		
F2	FLX	9	OM5<br>OM6<br>OM7	OM4<br>OM8	OF5<br>OF8	OF6<br>OF7		
F3	CTR	13	OM9<br>OM12	OM10<br>OM11	OF12<br>OF16	OF9<br>OF15	OF11<br>OF14	OF10<br>OF13<br>OF17
F4	CTR	6	OM13<br>OM14<br>OM15		OF18<br>OF19<br>OF20			
F5	FLX	None						
F6	FLX	None						
F7	CTR	None						
F8	CTR	15	OM16<br>OM17	OM20<br>OM21<br>OM25	OF21<br>OF23	OF22<br>OF24<br>OF25		
F9	FLX	8	OM29<br>OM30<br>OM31		OF27<br>OF29	OF26<br>OF28<br>OF30		
F10	FLX	dead						

Note. M = male, F= female, CTR = methylcellulose, FLX = fluoxetine, OM = male offspring, OF = female offspring

Figure S1

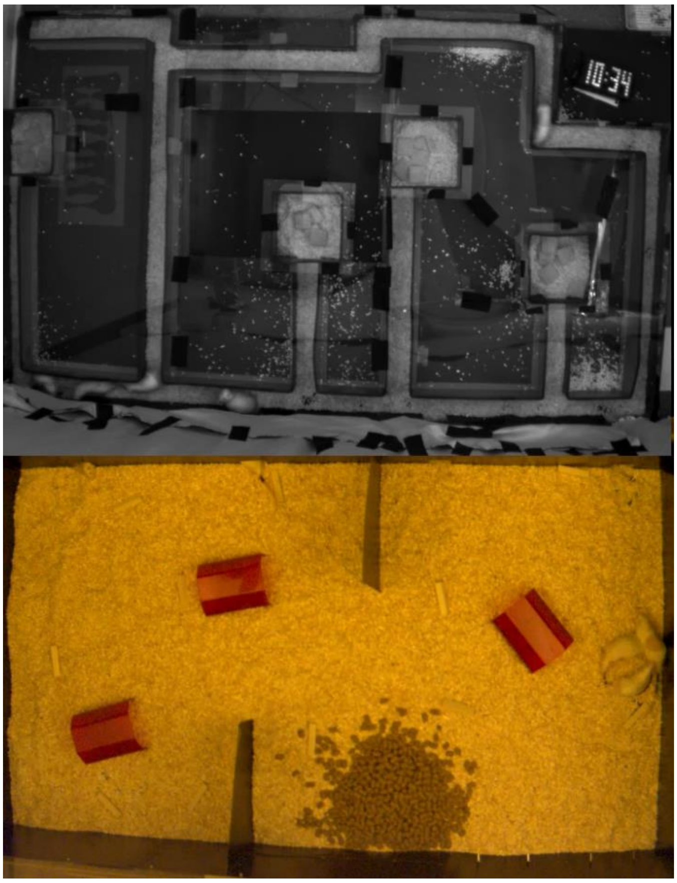

Figure S1. Picture of the whole seminatural environment

Table S2

Experimental Design

Colony	Male 1	Male 2	Male 3	Male 4	Female 1	Female 2	Female 3	Female 4
SNE1	OM1	OM5	OM9	OM13	OF2	OF5	OF12	OF21
SNE2	OM2	OM6	OM12	OM16	OF3	OF8	OF13	OF22
SNE3	OM3	OM7	OM15	OM17	OF1	OF6	OF15	OF24
SNE4	OM4	OM11	OM21	OM29	OF4	OF9	OF19	OF27
SNE5	OM8	OM10	OM20	OM30	OF7	OF14	OF20	OF26

Note. SNE = seminatural environment, OM = male offspring, OF female offspring

Table S3

Means and Standard Error for Duration of Behaviors

Behavior		10	20	30	40	50	60	Total
Socially active behaviors	CTR-females	80.5 ± 10.9	58.9 ± 6.1	61.4 ± 8.5	75.2 ± 9.5	69.2 ± 8.1	53.4 ± 5.8	398.6 ± 21.3
	FLX-females	89.0 ± 9.3	67.9 ± 9.0	65.9 ± 5.4	81.4 ± 9.5	86.4 ± 9.0	54.4 ± 10.5	445.1 ± 39.2
	CTR-males	97.7 ± 13.6	104.3 ± 23.6	120.6 ± 16.8	109.5 ± 17.2	110.3 ± 13.0	94.4 ± 16.9	636.5 ± 62.1
	FLX-males	79.7 ± 12.9	100.5 ± 25.3	91.2 ± 13.8	77.3 ± 10.9	106.0 ± 14.2	93.6 ± 13.0	548.0 ± 68.8
General activity	CTR-females	412.7 ± 11.0	**400.2 ± 7.4**	391.5 ± 11.6	391.4 ± 12.4	361.9 ± 12.7	388.3 ± 19.3	2346.0 ± 35.1
	FLX-females	391.5 ± 8.4	**372.7 ± 8.6**	394.9 ± 11.8	389.3 ± 5.7	368.3 ± 27.1	390.0 ± 8.0	2308.1 ± 51.2
	CTR-males	362.1 ± 28.6	339.4 ± 27.3	298.1 ± 18.0	**267.7 ± 13.9**	287.4 ± 28.9	**256.4 ± 19.4**	1810.3 ± 95.4
	FLX-males	383.1 ± 19.6	336.6 ± 24.0	325 ± 21.6	**327.5 ± 9.5**	323.9 ± 15.3	**308.3 ± 14.3**	2006.0 ± 66.1
Non-socially passive behaviors	CTR-females	16.4 ± 4.7	12.3 ± 3.3	14.3 ± 4.0	16.4 ± 4.4	36.8 ± 13.2	30.0 ± 10.0	568.1 ± 59.7
	FLX-females	12.7 ± 2.8	11.9 ± 2.1	15.6 ± 2.3	14.2 ± 5.0	25.3 ± 13.0	26.8 ± 4.5	614.7 ± 80.5
	CTR-males	21.1 ± 5.9	19.1 ± 5.1	27.9 ± 6.3	46.0 ± 14.1	39.4 ± 18.8	60.8 ± 16.8	273.7 ± 44.1
	FLX-males	18.5 ± 6.8	13.4 ± 3.1	17.8 ± 3.9	36.0 ± 11.6	14.5 ± 4.1	34.6 ± 10.2	163.0 ± 30.6
Socially passive behaviors	CTR-females	2.5 ± 1.4	6.3 ± 3.2	3.2 ± 1.1	4.2 ± 1.1	16.9 ± 8.5	5.1 ± 2.7	38.1 ± 9.3
	FLX-females	2.8 ± 1.5	3.9 ± 1.9	8.1 ± 3.4	4.9 ± 1.7	18.1 ± 10.6	3.8 ± 1.6	41.6 ± 14.2
	CTR-males	4.1 ± 2.2	3.3 ± 2.7	2.6 ± 1.6	15.4 ± 5.3	14.9 ± 8.2	19.3 ± 8.7	29.2 ± 10.0
	FLX-males	2.6 ± 1.3	2.5 ± 1.1	3.9 ± 1.3	7.3 ± 3.2	4.6 ± 1.4	7.7 ± 1.8	29.6 ± 6.7
Conflict behaviors	CTR-females	8.2 ± 2.6	5.9 ± 1.7	11.4 ± 4.1	14.1 ± 4.8	11.2 ± 3.3	8.1 ± 1.8	58.8 ± 12.0
	FLX-females	8.4 ± 2.0	12.6 ± 3.1	10.7 ± 3.0	7.9 ± 1.5	8.1 ± 2.5	10.9 ± 3.7	58.4 ± 9.8
	CTR-males	1.5 ± 0.9	1.9 ± 0.9	3.8 ± 1.7	9.3 ± 3.7	4.6 ± 2.6	19.5 ± 13.4	40.6 ± 16.5
	FLX-males	2.0 ± 1.1	4.2 ± 3.1	10.9 ± 4.0	16.5 ± 7.2	9.5 ± 2.2	8.7 ± 4.6	51.2 ± 13.2
Walking/running	CTR-females	**74.1 ± 8.1**	79.7 ± 14.7	**63.2 ± 6.2**	68.1 ± 7.7	65.4 ± 9.7	54.4 ± 7.9	**404.9 ± 29.4**
	FLX-females	**111.6 ± 10.9**	116.3 ± 14.7	**115.2 ± 18.0**	95.5 ± 10.5	102.2 ± 24.7	75.7 ± 13.1	**616.5 ± 80.1**
	CTR-males	157.3 ± 17.3	155.5 ± 13.1	131.8 ± 11.9	**143.4 ± 6.4**	**129.0 ± 14.6**	122.3 ± 6.7	**839.0 ± 40.7**
	FLX-males	176.7 ± 18.7	175.8 ± 14.0	162.8 ± 13.1	**186.4 ± 10.2**	**185.5 ± 16.6**	154.8 ± 13.7	**1041.6 ± 52.6**
Chasing	CTR-females	0.0 ± 0.0	0.0 ± 0.0	0.0 ± 0.0	0.3 ± 0.03	0.0 ± 0.0	0.0 ± 0.0	0.3 ± 0.3
	FLX-females	0.0 ± 0.0	0.0 ± 0.0	0.0 ± 0.0	0.0 ± 0.0	0.0 ± 0.0	0.0 ± 0.0	0.0 ± 0.0
	CTR-males	2.8 ± 1.8	0.2 ± 0.2	0.0 ± 0.0	0.0 ± 0.0	0.0 ± 0.0	0.0 ± 0.0	3.0 ± 2.0
	FLX-males	0.0 ± 0.0	0.3 ± 0.3	0.3 ± 0.3	3.6 ± 2.4	0.0 ± 0.0	1.0 ± 0.7	5.3 ± 3.2
Non-social exploration	CTR-females	**338.7 ± 16.2**	**320.5 ± 8.8**	**328.3 ± 10.5**	323.3 ± 18.0	296.4 ± 11.8	333.9 ± 23.1	**1941.1 ± 48.0**
	FLX-females	**279.9 ± 10.7**	**256.3 ± 11.7**	**279.6 ±14.6**	293.8 ± 13.1	266.1 ± 26.1	314.3 ± 15.4	**1691.5 ± 64.7**
	CTR-males	204.8 ± 36.1	183.9 ± 29.2	166.3 ± 16.9	124.3 ± 11.0	158.5 ± 22.5	134.0 ± 19.9	971.3 ± 89.5
	FLX-males	206.4 ± 31.6	160.9 ± 13.5	162.5 ± 13.9	141.0 ± 16.9	138.3 ± 14.6	153.5 ± 16.2	964.4 ± 72.0
Digging	CTR-females	5.5 ± 3.2	31.0 ± 11.6	26.0 ± 4.8	17.8 ± 5.6	14.7 ± 4.1	16.9 ± 4.8	111.8 ± 21.9
	FLX-females	12.6 ± 3.5	28.0 ± 7.6	32.2 ± 7.5	14.6 ± 2.2	15.7 ± 2.2	29.9 ± 8.1	133.1 ± 18.3
	CTR-males	0.8 ± 0.8	2.4 ± 1.7	16.7 ± 8.3	8.5 ± 7.0	3.0 ± 1.8	2.9 ± 1.9	34.2 ± 14.4
	FLX-males	4.7 ± 2.8	6.4 ± 3.0	13.3 ± 8.7	5.3 ± 4.0	2.9 ± 1.8	3.6 ± 2.2	33.9 ± 14.6
Resting/immobile alone	CTR-females	1.1 ± 0.7	0.0 ± 0.0	3.6 ± 3.3	0.5 ± 0.3	7.9 ± 6.4	8.8 ± 8.3	21.8 ± 14.7
	FLX-females	1.0 ± 1.0	0.3 ± 0.3	3.1 ± 1.9	4.0 ± 2.6	4.6 ± 3.8	11.8 ± 4.4	24.8 ± 9.2
	CTR-males	17.5 ± 6.4	16.4 ± 5.6	24.7 ± 6.6	34.9 ± 8.6	33.5 ± 17.5	57.1 ± 17.5	183.8 ± 35.4
	FLX-males	15.3 ± 6.6	11.8 ± 3.1	14.6 ± 4.2	32.2 ± 11.7	13.4 ± 4.1	29.1 ± 10.1	116.1 ± 27.6
Resting/immobile socially	CTR-females	0.0 ± 0.0	0.0 ± 0.0	0.0 ± 0.0	0.3 ± 0.3	0.8 ± 0.4	0.0 ± 0.0	1.1 ± 0.6
	FLX-females	0.0 ± 0.0	0.0 ± 0.0	0.2 ± 0.2	1.2 ± 0.7	6.4 ± 4.0	0.2 ± 0.2	7.9 ± 4.5

	CTR-males	2.0 ± 0.9	1.4 ± 0.8	0.4 ± 0.3	9.5 ± 4.9	14.1 ± 8.4	18.2 ± 9.0	45.5 ± 19.9
	FLX-males	2.4 ± 1.4	0.7 ± 0.5	2.4 ± 1.2	4.7 ± 2.9	2.8 ± 1.2	5.0 ± 1.5	18.1 ± 4.9
Hiding alone	CTR-females	15.3 ± 4.6	12.3 ± 3.3	10.7 ± 3.1	16.0 ± 4.5	28.8 ± 9.6	21.3 ± 5.7	104.3 ± 18.8
	FLX-females	11.7 ± 2.2	11.6 ± 2.2	12.5 ± 2.5	10.2 ± 3.9	20.7 ± 9.4	15.0 ± 4.1	81.7 ± 11.7
	CTR-males	3.5 ± 2.1	2.8 ± 1.1	3.2 ± 2.2	11.1 ± 10.1	5.9 ± 2.1	3.7 ± 2.2	30.2 ± 15.1
	FLX-males	3.2 ± 1.5	1.6 ± 0.9	3.2 ± 1.7	3.8 ± 1.4	1.1 ± 0.5	5.5 ± 3.2	18.4 ± 5.1
Hiding socially	CTR-females	2.5 ± 1.4	6.3 ± 3.2	3.2 ± 1.1	3.9 ± 1.2	16.1 ± 8.4	5.1 ± 2.7	37.1 ± 9.4
	FLX-females	2.8 ± 1.5	3.9 ± 1.9	7.9 ± 3.4	3.7 ± 1.9	11.8 ± 8.3	3.6 ± 1.6	33.7 ± 12.7
	CTR-males	2.1 ± 2.1	2.0 ± 2.0	2.3 ± 1.7	5.9 ± 2.5	0.8 ± 0.5	1.1 ± 0.8	14.1 ± 5.8
	FLX-males	1.9 ± 1.9	1.8 ± 1.2	1.5 ± 0.9	2.5 ± 1.5	1.8 ± 1.4	2.8 ± 1.2	10.5 ± 4.1
Following	CTR-females	8.2 ± 6.1	1.7 ± 1.3	1.7 ± 1.0	1.9 ± 1.4	1.3 ± 1.0	1.6 ± 1.3	16.5 ± 10.7
	FLX-females	4.6 ± 1.9	2.3 ± 1.4	1.6 ± 0.6	1.8 ± 1.2	1.5 ± 1.0	1.3 ± 0.9	13.0 ± 5.0
	CTR-males	33.3 ± 9.0	30.8 ± 7.8	36.5 ± 10.3	48.8 ± 12.7	39.7 ± 8.5	36.5 ± 10.5	225.5 ± 47.9
	FLX-males	30.7 ± 5.6	36.5 ± 6.7	34.4 ± 5.3	26.1 ± 6.0	32.2 ± 6.5	30.6 ± 5.9	189.7 ± 26.4
Allogrooming	CTR-females	0.0 ± 0.0	0.0 ± 0.0	0.0 ± 0.0	1.0 ± 0.8	1.4 ± 1.1	0.2 ± 0.2	2.6 ± 1.1
	FLX-females	0.0 ± 0.0	0.3 ± 0.3	0.0 ± 0.0	0.0 ± 0.0	0.9 ± 0.9	0.2 ± 0.2	1.3 ± 0.9
	CTR-males	1.5 ± 1.0	3.7 ± 3.0	1.0 ± 1.0	2.3 ± 1.6	1.8 ± 1.5	4.4 ± 4.4	14.5 ± 12.1
	FLX-males	1.2 ± 0.8	1.2 ± 1.2	0.0 ± 0.0	0.2 ± 0.2	0.7 ± 0.4	0.2 ± 0.2	3.5 ± 2.0
Sniffing anogenitally	CTR-females	35.9 ± 9.0	20.7 ± 2.3	20.1 ± 5.8	19.0 ± 5.4	20.9 ± 3.6	14.8 ± 2.2	131.4 ± 8.3
	FLX-females	39.7 ± 5.4	27.7 ± 5.8	23.5 ± 5.1	29.6 ± 7.7	25.2 ± 5.1	17.7 ± 3.7	163.1 ± 12.3
	CTR-males	52.9 ± 12.1	64.1 ± 19.7	85.9 ± 14.7	68.7 ± 13.2	68.1 ± 9.6	52.5 ± 11.6	391.9 ± 44.6
	FLX-males	38.2 ± 10.7	70.8 ± 21.1	62.3 ± 14.4	43.8 ± 10.4	66.3 ± 13.2	61.1 ± 8.9	342.3 ± 58.3
Sniffing nose-to-nose	CTR-females	13.5 ± 5.0	10.5 ± 3.8	16.3 ± 8.2	21.4 ± 4.2	18.1 ± 1.7	15.7 ± 2.6	95.5 ± 21.9
	FLX-females	10.4 ± 2.3	9.4 ± 0.8	14.5 ± 2.2	18.3 ± 3.1	27.5 ± 10.8	10.3 ± 2.0	90.6 ± 15.4
	CTR-males	3.5 ± 0.7	3.6 ± 1.1	4.6 ± 1.5	7.5 ± 2.2	8.8 ± 3.7	8.8 ± 1.8	36.7 ± 9.0
	FLX-males	3.6 ± 1.2	2.2 ± 1.1	3.5 ± 1.0	5.0 ± 0.9	6.4 ± 2.3	5.7 ± 1.3	26.4 ± 5.6
All sniffing	CTR-females	80.5 ± 10.9	58.9 ± 6.1	61.4 ± 8.5	74.3 ± 9.5	67.8 ± 7.4	53.1 ± 5.8	360.0 ± 22.2
	FLX-females	89.0 ± 9.5	67.6 ± 8.8	65.9 ± 5.4	81.4 ± 9.5	85.6 ± 9.2	54.3 ± 5.8	443.8 ± 39.2
	CTR-males	96.2 ± 14.4	100.6 ± 23.4	119.6 ± 16.5	107.2 ± 16.7	108.6 ± 12.4	90.0 ± 14.3	622.0 ± 54.8
	FLX-males	78.5 ± 13.1	99.3 ± 24.4	91.2 ± 13.8	77.1 ± 11.0	105.3 ± 14.4	93.4 ± 13.0	544.5 ± 68.8
Sniffing body	CTR-females	31.2 ± 5.3	27.7 ± 5.1	25.0 ± 3.9	33.8 ± 6.4	28.8 ± 5.0	22.6 ± 3.0	169.1 ± 21.6
	FLX-females	39.1 ± 6.0	30.5 ± 3.7	27.9 ± 3.9	33.5 ± 3.5	32.9 ± 5.4	26.2 ± 6.3	190.1 ± 21.7
	CTR-males	40.0 ± 3.0	33.0 ± 4.8	29.2 ± 6.5	31.0 ± 4.6	31.7 ± 6.7	28.7 ± 4.1	193.4 ± 12.5
	FLX-males	36.8 ± 4.9	26.3 ± 4.1	25.3 ± 2.7	28.2 ± 4.8	32.6 ± 3.0	26.7 ± 4.9	175.7 ± 18.6
Fighting	CTR-females	8.2 ± 2.6	5.9 ± 1.7	11.4 ± 4.1	14.1 ± 4.8	11.2 ± 3.3	8.1 ± 1.8	58.8 ± 12.0
	FLX-females	8.4 ± 2.0	12.6 ± 3.1	10.7 ± 3.0	7.8 ± 1.5	8.1 ± 2.5	10.9 ± 3.7	58.4 ± 9.8
	CTR-males	1.5 ± 0.9	1.9 ± 0.9	3.8 ± 1.7	8.3 ± 3.3	4.2 ± 2.5	5.8 ± 2.1	25.5 ± 6.0
	FLX-males	2.0 ± 1.1	4.2 ± 3.1	10.9 ± 4.0	15.1 ± 6.7	7.2 ± 2.0	5.8 ± 2.8	45.2 ± 11.9
Nose-off	CTR-females	0.0 ± 0.0	0.0 ± 0.0	0.0 ± 0.0	0.0 ± 0.0	0.0 ± 0.0	0.0 ± 0.0	0.0 ± 0.0
	FLX-females	0.0 ± 0.0	0.0 ± 0.0	0.0 ± 0.0	0.1 ± 0.1	0.0 ± 0.0	0.0 ± 0.0	0.1 ± 0.1
	CTR-males	0.0 ± 0.0	0.0 ± 0.0	0.0 ± 0.0	1.9 ± 0.6	0.4 ± 0.2	13.7 ± 12.4	15.1 ± 12.7
	FLX-males	0.0 ± 0.0	0.0 ± 0.0	0.1 ± 01	1.3 ± 0.6	2.3 ± 1.3	2.9 ± 1.9	6.0 ± 2.4
Self-grooming	CTR-females	8.5 ± 3.2	5.3 ± 1.6	20.2 ± 5.8	12.1 ± 3.4	19.0 ± 7.6	29.1 ± 10.9	94.2 ± 12.5
	FLX-females	3.4 ± 1.1	6.9 ± 1.3	8.8 ± 2.4	15.0 ± 5.1	11.2 ± 3.1	25.3 ± 7.4	70.5 ± 6.8
	CTR-males	1.0 ± 0.6	7.3 ± 4.4	3.4 ± 2.3	9.6 ± 5.0	21.9 ± 11.7	22.3 ± 9.5	65.2 ± 25.3
	FLX-males	1.2 ± 0.8	2.6 ± 1.1	16.7 ± 5.8	19.0 ± 9.5	10.3 ± 5.8	15.1 ± 6.0	64.6 ± 16.5
Freezing	CTR-females	0.0 ± 0.0	0.1 ± 0.1	0.4 ± 0.2	5.3 ± 3.2	1.6 ± 0.8	0.4 ±0.2	7.7 ± 3.6
	FLX-females	0.0 ± 0.0	0.1 ± 0.1	0.6 ± 0.4	6.9 ± 4.1	2.9 ± 1.1	0.9 ± 0.4	11.5 ± 4.6
	CTR-males	0.9 ± 0.6	0.0 ± 0.0	0.2 ± 0.2	3.6 ± 1.9	0.5 ± 0.3	0.9 ± 0.5	6.0 ± 2.3
	FLX-males	0.2 ± 0.2	0.0 ± 0.0	0.6 ± 0.6	4.0 ± 2.6	0.3 ± 0.2	7.1 ± 3.0	12.1 ± 3.4
Rearing supported	CTR-females	41.7 ± 5.9	62.5 ± 6.2	52.3 ± 8.7	47.7 ± 9.3	53.8 ± 7.6	58.2 ± 8.8	316.2 ± 25.3
	FLX-females	60.3 ± 8.7	80.8 ± 9.2	51.3 ± 5.2	51.4 ± 10.4	50.1 ± 5.9	49.1 ± 6.3	343.2 ± 27.6
	CTR-males	20.9 ± 3.0	37.7 ± 8.1	26.8 ± 4.2	24.8 ± 6.6	16.7 ± 4.9	19.5 ± 4.3	145.9 ± 17.5
	FLX-males	20.9 ± 3.4	36.4 ± 7.5	26.1 ± 6.1	16.9 ± 5.4	16.0 ± 4.7	13.5 ± 2.1	129.8 ± 18.4
Rearing unsupported	CTR-females	0.9 ± 0.2	1.9 ± 1.2	1.8 ± 0.7	1.5 ± 0.5	3.6 ± 1.2	1.9 ± 0.6	11.4 ± 3.1
	FLX-females	0.7 ± 0.3	2.5 ± 1.4	2.1 ± 1.9	2.1 ± 0.9	3.9 ± 1.2	3.0 ± 1.2	14.3 ± 2.6
	CTR-males	0.5 ± 0.4	0.5 ± 0.3	3.9 ± 1.8	4.6 ± 1.9	1.7 ± 0.8	3.8 ± 1.8	15.0 ± 5.6
	FLX-males	0.3 ± 0.3	1.7 ± 1.3	4.4 ± 1.8	1.6 ± 0.7	1.6 ± 0.7	9.7 ± 4.0	19.1 ± 3.6

Note. The data represent the time spent (s) performing all behaviors measured within the six time-bins. Data are shown in mean ± standard error of the mean.

Table S4

Means and Standard Error for Frequency of Behaviors

Behavior		10	20	30	40	50	60	Total
Socially active behaviors	CTR-females	59.0 ± 10.7	55.1 ± 7.0	49.4 ± 4.9	66.9 ± 9.3	56.5 ± 5.3	48.5 ± 3.6	335.4 ± 32.0
	FLX-females	69.5 ± 6.6	68.1 ± 8.7	62.8 ± 5.1	67.4 ± 5.8	66.5 ± 9.2	48.3 ± 8.2	382.6 ± 32.5
	CTR-males	47.9 ± 8.7	44.6 ± 7.0	54.0 ± 8.5	51.1 ± 8.1	50.5 ± 5.1	51.0 ± 9.2	299.0 ± 36.5
	FLX-males	42.4 ± 6.7	45.1 ± 8.7	46.9 ± 3.9	47.0 ± 5.3	61.4 ± 6.6	51.3 ± 4.9	294.0 ± 31.6
General activity	CTR-females	159.0 ± 24.1	175.5 ± 22.1	171.4 ± 21.9	177.4 ± 18.5	162.6 ± 17.6	155.9 ± 9.3	1001.8 ± 93.2
	FLX-females	204.8 ± 17.8	224.3 ± 14.9	213.8 ± 9.3	207.4 ± 15.4	189.8 ± 16.9	169.3 ± 22.8	1209.6 ± 66.1
	CTR-males	92.5 ± 6.4	98.6 ± 9.6	88.8 ± 10.0	83.9 ± 8.8	76.8 ± 8.3	75.5 ± 9.4	516.0 ± 44.2
	FLX-males	97.3 ± 6.9	99.3 ± 4.9	100.5 ± 5.3	98.3 ± 5.2	97.6 ± 6.1	97.5 ± 5.3	590.3 ± 13.6
Non-socially passive behaviors	CTR-females	5.5 ± 0.3	5.6 ± 1.0	5.6 ± 1.6	6.6 ± 1.5	7.8 ± 2.0	8.0 ± 2.3	51.6 ± 8.1
	FLX-females	5.5 ± 1.0	5.5 ± 0.9	7.4 ± 1.3	6.4 ± 1.5	7.3 ± 1.6	8.0 ± 1.7	55.0 ± 6.2
	CTR-males	3.9 ± 0.8	3.9 ± 0.6	4.1 ± 1.1	6.3 ± 1.6	7.0 ± 2.6	8.0 ± 1.5	44.6 ± 5.0
	FLX-males	4.3 ± 1.6	2.9 ± 0.6	4.0 ± 0.7	6.5 ± 1.7	3.9 ± 1.0	7.3 ± 2.0	35.5 ± 5.9
Socially passive behaviors	CTR-females	1.0 ± 0.3	2.0 ± 0.6	1.4 ± 0.5	1.9 ± 0.4	3.8 ± 1.2	2.0 ± 0.9	12.0 ± 2.7
	FLX-females	1.1 ± 0.4	1.8 ± 0.7	2.5 ± 0.8	1.5 ± 0.4	4.5 ± 1.4	1.6 ± 0.7	13.0 ± 2.4
	CTR-males	1.3 ± 0.8	0.9 ± 0.6	0.5 ± 0.2	2.4 ± 0.7	3.6 ± 1.6	2.9 ± 1.1	11.5 ± 3.1
	FLX-males	0.5 ± 1.2	0.6 ± 0.3	1.0 ± 0.3	1.6 ± 0.7	1.3 ± 0.3	1.8 ± 0.3	6.8 ± 0.9
Conflict behaviors	CTR-females	3.8 ± 0.9	5.0 ± 1.2	4.9 ± 1.2	7.8 ± 1.0	5.8 ± 1.9	5.0 ± 0.8	32.1 ± 3.9
	FLX-females	5.4 ± 1.5	6.5 ± 1.6	6.5 ± 1.3	6.5 ± 1.1	4.9 ± 1.1	5.0 ± 1.8	34.8 ± 5.2
	CTR-males	0.8 ± 0.4	1.4 ± 0.5	1.6 ± 0.5	3.1 ± 1.1	2.6 ± 0.9	1.9 ± 0.8	11.4 ± 2.7
	FLX-males	0.9 ± 0.5	1.1 ± 0.5	2.8 ± 0.5	6.3 ± 2.7	3.9 ± 1.1	2.4 ± 0.9	17.1 ± 4.0
Walking/running	CTR-females	**44.4 ± 7.6**	**52.6 ± 8.5**	**51.1 ± 8.1**	**50.4 ± 6.5**	46.1 ± 7.2	46.0 ± 4.5	**290.6 ± 28.3**
	FLX-females	**75.4 ± 3.0**	**88.5 ± 8.9**	**81.6 ± 4.7**	**73.3 ± 5.3**	71.6 ± 9.9	56.6 ± 10.7	**447.0 ± 23.0**
	CTR-males	52.5 ± 5.4	57.8 ± 6.9	51.8 ± 7.3	53.3 ± 6.2	46.6 ± 5.1	**48.9 ± 6.9**	310.8 ± 29.3
	FLX-males	54.6 ± 6.4	61.6 ± 4.3	61.5 ± 4.0	65.4 ± 5.1	64.4 ± 5.8	**59.1 ± 4.5**	366.4 ± 18.5
Chasing	CTR-females	0.0 ± 0.0	0.0 ± 0.0	0.0 ± 0.0	0.1 ± 0.1	0.0 ± 0.0	0.0 ± 0.0	0.1 ± 0.1
	FLX-females	0.0 ± 0.0	0.0 ± 0.0	0.0 ± 0.0	0.0 ± 0.0	0.0 ± 0.0	0.0 ± 0.0	0.0 ± 0.0
	CTR-males	1.8 ± 1.3	0.1 ± 0.1	0.0 ± 0.0	0.0 ± 0.0	0.0 ± 0.0	0.0 ± 0.0	1.9 ± 1.4
	FLX-males	0.0 ± 0.0	0.1 ± 0.1	0.1 ± 0.1	1.4 ± 0.9	0.0 ± 0.0	0.5 ± 0.4	2.1 ± 1.3
Non-social exploration	CTR-females	114.6 ± 17.0	122.9 ± 15.8	120.3 ± 14.4	127.0 ± 13.9	116.5 ± 11.2	109.9 ± 8.9	711.1 ± 71.1
	FLX-females	129.4 ± 15.0	135.8 ± 12.3	132.1 ± 12.0	134.1 ± 12.9	118.1 ± 13.9	112.6 ± 13.7	762.6 ± 65.0
	CTR-males	40.0 ± 3.8	40.9 ± 5.8	37.0 ± 3.8	30.6 ± 4.1	30.1 ± 4.9	**26.6 ± 3.4**	205.3 ± 21.7
	FLX-males	42.6 ± 3.3	37.6 ± 2.8	39.0 ± 2.7	32.9 ± 2.9	33.3 ± 3.3	**38.4 ± 4.2**	223.9 ± 13.7
Digging	CTR-females	2.9 ± 1.4	6.4 ± 1.9	6.1 ± 1.5	3.5 ± 1.1	3.4 ± 0.7	5.4 ± 1.1	27.6 ± 5.2
	FLX-females	5.0 ± 1.7	8.6 ± 1.7	8.8 ± 2.2	2.5 ± 0.7	3.4 ± 0.5	6.5 ± 1.3	35.8 ± 4.8
	CTR-males	0.3 ± 0.3	0.6 ± 0.4	0.9 ± 0.4	1.4 ± 1.0	0.9 ± 0.5	0.8 ± 0.5	4.8 ± 2.1
	FLX-males	0.9 ± 0.5	1.6 ± 0.7	2.5 ± 1.4	1.4 ± 0.8	0.5 ± 0.3	0.4 ± 0.2	7.1 ± 2.8
Resting/immobile alone	CTR-females	0.4 ± 0.3	0.0 ± 0.0	0.4 ± 0.3	0.5 ± 0.3	0.9 ± 0.4	0.4 ± 0.3	2.5 ± 1.0
	FLX-females	0.3 ± 0.3	0.1 ± 0.1	1.0 ± 0.6	1.5 ± 0.9	1.1 ± 0.5	2.1 ± 0.9	6.1 ± 2.2
	CTR-males	3.1 ± 0.9	3.4 ± 0.6	3.6 ± 1.1	5.3 ± 1.1	5.6 ± 2.2	7.4 ± 1.6	28.4 ± 4.2
	FLX-males	3.4 ± 1.5	2.3 ± 0.6	3.3 ± 0.7	5.5 ± 1.6	3.4 ± 1.0	6.0 ± 1.9	23.8 ± 5.1
Resting/immobile socially	CTR-females	0.0 ± 0.0	0.0 ± 0.0	0.0 ± 0.0	0.1 ± 0.1	0.4 ± 0.2	0.0 ± 0.0	0.5 ± 0.3
	FLX-females	0.0 ± 0.0	0.0 ± 0.0	0.1 ± 0.1	0.5 ± 0.3	1.3 ± 0.7	0.1 ± 0.1	2.0 ± 0.8
	CTR-males	0.5 ± 0.2	0.5 ± 0.3	0.3 ± 0.2	1.4 ± 0.7	3.3 ± 1.7	2.5 ± 1.2	8.4 ± 2.9
	FLX-males	0.4 ± 0.2	0.3 ± 0.2	0.5 ± 0.2	1.0 ± 0.6	0.9 ± 0.4	1.1 ± 0.2	4.1 ± 1.0
Hiding alone	CTR-females	5.1 ± 0.5	5.6 ± 1.0	5.3 ± 1.6	6.1 ± 1.6	6.9 ± 1.9	7.6 ± 2.2	36.6 ± 5.9
	FLX-females	5.3 ± 1.0	5.4 ± 0.9	6.4 ± 1.3	4.9 ± 1.4	6.1 ± 1.2	5.9 ± 1.7	33.9 ± 4.1
	CTR-males	0.8 ± 0.4	0.5 ± 0.2	0.5 ± 0.3	1.0 ± 0.7	1.4 ± 0.5	0.6 ± 0.3	4.8 ± 1.5
	FLX-males	0.9 ± 0.4	0.6 ± 0.3	0.8 ± 0.4	1.0 ± 0.3	0.5 ± 0.2	1.3 ± 0.7	5.0 ± 1.4
Hiding socially	CTR-females	1.0 ± 0.3	2.0 ± 0.6	1.4 ± 0.5	1.8 ± 0.5	3.4 ± 1.1	2.0 ± 0.9	11.5 ± 2.8
	FLX-females	1.1 ± 0.4	1.8 ± 0.7	2.4 ± 0.8	1.0 ± 0.4	3.3 ± 1.2	1.5 ± 0.7	11.0 ± 2.4
	CTR-males	0.8 ± 0.8	0.4 ± 0.4	0.3 ± 0.2	1.0 ± 0.3	0.4 ± 0.3	0.4 ± 0.3	3.1 ± 1.5
	FLX-males	0.1 ± 0.1	0.4 ± 0.3	0.5 ± 0.3	0.6 ± 0.3	0.4 ± 0.3	0.6 ± 0.3	2.6 ± 1.0
Following	CTR-females	2.0 ± 0.8	0.8 ± 0.5	0.6 ± 0.3	0.9 ± 0.4	0.6 ± 0.4	0.8 ± 0.5	5.6 ± 2.1
	FLX-females	1.9 ± 0.7	1.3 ± 0.6	1.1 ± 0.6	0.8 ± 0.5	1.1 ± 0.7	0.9 ± 0.5	7.0 ± 2.5
	CTR-males	16.6 ± 4.9	14.0 ± 3.2	17.9 ± 4.9	20.1 ± 5.3	16.9 ± 4.0	15.3 ± 5.1	100.8 ± 22.7
	FLX-males	12.9 ± 2.9	15.1 ± 3.1	14.0 ± 1.8	12.0 ± 2.5	13.4 ± 2.4	13.6 ± 2.8	81.1 ± 11.3

Allogrooming	CTR-females	0.0 ± 0.0	0.0 ± 0.0	0.0 ± 0.0	0.4 ± 0.3	0.3 ± 0.2	0.1 ± 0.1	0.8 ± 0.3
	FLX-females	0.0 ± 0.0	0.1 ± 0.1	0.0 ± 0.0	0.0 ± 0.0	0.1 ± 0.1	0.1 ± 0.1	0.4 ± 0.2
	CTR-males	0.4 ± 0.3	0.6 ± 0.5	0.3 ± 0.3	0.3 ± 0.2	0.3 ± 0.2	0.8 ± 0.8	2.5 ± 2.0
	FLX-males	0.4 ± 0.3	0.4 ± 0.4	0.0 ± 0.0	0.1 ± 0.1	0.4 ± 0.2	0.1 ± 0.1	1.4 ± 0.8
Sniffing anogenitally	CTR-females	20.8 ± 5.1	15.6 ± 1.6	13.1 ± 3.1	13.6 ± 2.6	12.6 ± 1.6	9.9 ± 1.0	85.6 ± 7.0
	FLX-females	22.9 ± 2.5	20.3 ± 3.4	17.1 ± 2.8	16.6 ± 2.8	15.8 ± 3.0	11.8 ± 2.3	104.4 ± 7.0
	CTR-males	19.5 ± 5.2	20.1 ± 4.4	28.0 ± 5.8	23.3 ± 5.5	21.9 ± 3.9	20.4 ± 5.8	133.0 ± 22.8
	FLX-males	14.4 ± 3.4	21.5 ± 5.4	20.8 ± 3.4	16.3 ± 3.1	22.9 ± 3.4	20.5 ± 2.8	116.3 ± 17.7
Sniffing nose-to-nose	CTR-females	11.4 ± 1.9	11.5 ± 2.1	12.6 ± 1.8	21.1 ± 2.7	16.6 ± 1.1	16.9 ± 2.2	85.6 ± 7.0
	FLX-females	11.5 ± 1.1	12.1 ± 1.2	15.4 ± 1.4	19.8 ± 3.3	18.0 ± 1.7	12.1 ± 1.8	104.4 ± 7.0
	CTR-males	4.4 ± 0.8	4.8 ± 1.3	4.5 ± 1.1	6.6 ± 1.3	8.5 ± 2.5	8.6 ± 0.9	133.0 ± 22.8
	FLX-males	3.9 ± 1.0	3.3 ± 1.2	4.1 ± 1.0	6.4 ± 1.0	7.5 ± 1.9	7.0 ± 1.2	116.3 ± 17.7
All sniffing	CTR-females	59.0 ± 10.7	55.1 ± 7.0	49.4 ± 4.9	66.5 ± 9.3	56.3 ± 5.2	48.4 ± 3.6	90.1 ± 9.4
	FLX-females	69.5 ± 6.6	68.0 ± 8.6	62.8 ± 5.1	67.4 ± 5.8	66.4 ± 9.2	48.1 ± 8.2	89.0 ± 6.8
	CTR-males	47.5 ± 8.6	44.0 ± 6.7	53.8 ± 8.4	50.9 ± 8.0	50.3 ± 5.1	50.3 ± 8.7	37.4 ± 6.0
	FLX-males	42.0 ± 6.6	44.8 ± 8.5	46.9 ± 3.9	46.9 ± 5.3	61.0 ± 6.6	51.1 ± 4.9	32.1 ± 5.2
Sniffing body	CTR-females	26.9 ± 5.8	28.0 ± 5.6	23.6 ± 4.1	31.8 ± 6.4	27.0 ± 3.8	21.6 ± 2.3	158.9 ± 24.6
	FLX-females	35.1 ± 5.3	35.6 ± 4.9	30.3 ± 4.1	31.0 ± 3.2	32.6 ± 5.7	24.3 ± 5.1	188.9 ± 22.8
	CTR-males	23.6 ± 3.1	19.1 ± 1.6	21.3 ± 3.5	21.0 ± 3.2	**19.9 ± 2.3**	21.3 ± 2.8	126.1 ± 9.9
	FLX-males	23.8 ± 4.4	20.0 ± 3.1	22.0 ± 1.9	24.3 ± 3.5	**30.6 ± 3.3**	23.6 ± 2.6	144.3 ± 14.7
Fighting	CTR-females	3.8 ± 0.9	5.0 ± 1.2	4.9 ± 1.2	7.8 ± 1.0	5.8 ± 1.9	5.0 ± 0.8	32.1 ± 3.9
	FLX-females	5.4 ± 1.5	6.5 ± 1.6	6.5 ± 1.3	6.4 ± 1.1	4.9 ± 1.1	5.0 ± 1.8	34.6 ± 5.2
	CTR-males	0.8 ± 0.4	1.4 ± 0.5	1.6 ± 0.5	2.4 ± 0.7	2.3 ± 0.8	1.3 ± 0.5	9.6 ± 1.9
	FLX-males	0.9 ± 0.5	1.1 ± 0.5	2.6 ± 0.5	5.1 ± 2.2	3.1 ± 0.9	1.8 ± 0.7	14.6 ± 3.4
Nose-off	CTR-females	0.0 ± 0.0	0.0 ± 0.0	0.0 ± 0.0	0.0 ± 0.0	0.0 ± 0.0	0.0 ± 0.0	0.0 ± 0.0
	FLX-females	0.0 ± 0.0	0.0 ± 0.0	0.0 ± 0.0	0.1 ± 0.1	0.0 ± 0.0	0.0 ± 0.0	0.1 ± 0.1
	CTR-males	0.0 ± 0.0	0.0 ± 0.0	0.0 ± 0.0	0.8 ± 0.5	0.4 ± 0.2	0.6 ± 0.4	1.8 ± 0.9
	FLX-males	0.0 ± 0.0	0.0 ± 0.0	0.1 ± 0.1	1.1 ± 0.5	0.8 ± 0.3	0.6 ± 0.4	2.5 ± 0.8
Self-grooming	CTR-females	2.5 ± 0.5	3.0 ± 1.1	4.5 ± 1.0	4.1 ± 1.0	5.0 ± 1.4	5.5 ± 1.2	24.6 ± 3.8
	FLX-females	2.3 ± 0.7	3.3 ± 0.8	3.6 ± 0.5	3.5 ± 1.0	3.0 ± 0.9	6.4 ± 1.6	22.0 ± 2.5
	CTR-males	0.4 ± 0.3	0.9 ± 0.3	0.6 ± 0.4	1.4 ± 0.7	1.3 ± 0.4	2.0 ± 0.6	6.5 ± 1.5
	FLX-males	0.3 ± 0.2	0.6 ± 0.3	1.9 ± 0.6	2.4 ± 1.3	1.3 ± 0.5	1.5 ± 0.5	7.9 ± 1.7
Freezing	CTR-females	0.0 ± 0.0	0.1 ± 0.1	0.4 ± 0.3	2.6 ± 1.6	1.0 ± 0.6	0.5 ± 0.3	4.6 ± 2.4
	FLX-females	0.0 ± 0.0	0.1 ± 0.1	0.6 ± 0.4	2.1 ± 1.0	1.6 ± 0.5	1.0 ± 0.3	5.5 ± 1.6
	CTR-males	0.3 ± 0.2	0.0 ± 0.0	0.3 ± 0.3	1.0 ± 0.5	0.3 ± 0.2	0.4 ± 0.2	2.1 ± 0.7
	FLX-males	0.1 ± 0.1	0.0 ± 0.0	0.3 ± 0.3	0.8 ± 0.4	0.3 ± 0.2	1.3 ± 0.4	2.6 ± 0.6
Rearing supported	CTR-females	25.6 ± 5.3	32.0 ± 5.0	27.1 ± 4.4	25.9 ± 4.6	29.8 ± 3.3	30.1 ± 3.7	170.5 ± 17.2
	FLX-females	34.9 ± 4.7	40.1 ± 3.7	27.1 ± 2.1	27.4 ± 4.8	24.8 ± 4.1	27.0 ± 3.5	181.3 ± 14.6
	CTR-males	10.3 ± 2.1	16.5 ± 2.5	12.6 ± 2.4	11.9 ± 3.0	8.9 ± 1.9	9.3 ± 2.6	69.5 ± 9.1
	FLX-males	10.9 ± 2.7	16.3 ± 3.2	12.4 ± 2.4	8.8 ± 2.1	8.0 ± 2.0	8.0 ± 1.3	64.6 ± 7.7
Rearing unsupported	CTR-females	1.3 ± 0.3	1.4 ± 0.6	1.6 ± 0.4	1.1 ± 0.3	2.8 ± 0.8	1.5 ± 0.6	9.6 ± 1.6
	FLX-females	0.9 ± 0.3	2.3 ± 1.0	2.6 ± 2.1	2.5 ± 1.2	3.4 ± 0.9	2.0 ± 0.7	13.6 ± 2.8
	CTR-males	0.5 ± 0.3	0.4 ± 0.2	2.4 ± 1.1	2.8 ± 1.0	1.0 ± 0.4	2.4 ± 1.1	9.4 ± 3.4
	FLX-males	0.1 ± 0.1	1.1 ± 0.7	3.3 ± 1.2	1.0 ± 0.4	1.0 ± 0.4	4.9 ± 2.2	11.4 ± 2.4

Note. The data represent the number of instances performing all behaviors measured within the six time-bins. Data are shown in mean ± standard error of the mean.
